# Antiproliferative effects of ruthenium-based nucleolipidic nanoaggregates in human models of breast cancer *in vitro*: insights into their mode of action

**DOI:** 10.1038/srep45236

**Published:** 2017-03-28

**Authors:** Carlo Irace, Gabriella Misso, Antonella Capuozzo, Marialuisa Piccolo, Claudia Riccardi, Alessandra Luchini, Michele Caraglia, Luigi Paduano, Daniela Montesarchio, Rita Santamaria

**Affiliations:** 1 Department of Pharmacy, University of Naples “Federico II”, Via D. Montesano 49, 80131-Naples, Italy; 2 Department of Biochemistry, Biophysics and General Pathology, University of Campania “Luigi Vanvitelli”, Via L. De Crecchio 7, 80138-Naples, Italy; 3 Department of Chemical Sciences, University of Naples “Federico II”, Via Cintia 21, 80126-Naples, Italy; 4 Institut Laue-Langevin, 71 Avenue des Martyrs, 38000, Grenoble, France; 5 CSGI - Consorzio Sistemi a Grande Interfase, Department of Chemistry, University of Florence, Via della Lastruccia 3, 50019-Sesto Fiorentino (FI) Italy.

## Abstract

Looking for new metal-based anticancer treatments, in recent years many ruthenium complexes have been proposed as effective and safe potential drugs. In this context we have recently developed a novel approach for the *in vivo* delivery of Ru(III) complexes, preparing stable ruthenium-based nucleolipidic nanoaggregates endowed with significant antiproliferative activity. Herein we describe the cellular response to our ruthenium-containing formulations in selected models of human breast cancer. By *in vitro* bioscreens in the context of preclinical studies, we have focused on their ability to inhibit breast cancer cell proliferation by the activation of the intrinsic apoptotic pathway, possibly *via* mitochondrial perturbations involving Bcl-2 family members and predisposing to programmed cell death. In addition, the most efficient ruthenium-containing cationic nanoaggregates we have hitherto developed are able to elicit both extrinsic and intrinsic apoptosis, as well as autophagy. To limit chemoresistance and counteract uncontrolled proliferation, multiple cell death pathways activation by metal-based chemotherapeutics is a challenging, yet very promising strategy for targeted therapy development in aggressive cancer diseases, such as triple-negative breast cancer with limited treatment options. These outcomes provide valuable, original knowledge on ruthenium-based candidate drugs and new insights for future optimized cancer treatment protocols.

Metallodrugs exhibit a wide range of interesting biological activities, including transition metal-based compounds proved to be effective anticancer agents^1^,^2^,^3^. Cisplatin and its analogues have hitherto dominated the field of inorganic compounds endowed with high relevance in cancer treatment, so that platinum-based drugs are currently the most widely used chemotherapeutics^4^. Activation of cellular apoptosis triggered by DNA targeting seems to be the main determinant in causing bioactivity of cisplatin and its derivatives^5^. Based on the success of platinum-based anticancer drugs and aiming at overcoming the drawbacks related to their clinical use - i.e. high general toxicity, chemoresistance and inactivity against some cancer types - since the last decades a variety of novel metal-based chemotherapeutics are being investigated^6^,^7^. Within the intense search for more effective cytotoxic metallodrugs, ruthenium complexes have emerged among the most promising alternatives to platinum-based clinically validated anticancer agents^8^. This accounts for the large amount of current reports on novel candidate drugs investigated within the frame of a potential “ruthenotherapy”. Indeed, a number of ruthenium complexes, which exhibit interesting physico-chemical and biochemical properties, associated with low toxicity profiles, have been recently identified and intensively studied^9^,^10^. Many ruthenium complexes have in fact shown selective bioactivity, as well as the ability to overcome the resistance encountered with platinum-based drugs, ranking them as strong antitumoral candidates in a rational drug discovery approach^11^. Among these, ruthenium(III) compounds behave as valid prodrugs with somehow limited side-effects^11^, being likely activated to the more reactive and cytotoxic ruthenium(II) derivatives within the reducing microenvironment of solid tumours^12^. Along with ligands release and/or substitution - which occurs rapidly under physiological conditions *in vitro* and *in vivo* - the biological reduction of ruthenium(III) complexes is a possible process, especially in high proliferating cells, thereby promoting a unique activation process of this kind of metal-based drug in tumour tissues^12^. Nevertheless, in the general uncertainty concerning their mechanism of action, it cannot be excluded that Ru(III) complexes, properly transported into tumour cells, can interact in their original redox status with potential molecular targets. Furthermore, the possibility to be transported by the transferrin/transferrin receptor (Tf/TfR) network in place of iron, might allow for a natural ruthenium accumulation within cancer cells, typically requiring high iron amounts to accommodate for their rapid proliferation^13^,^14^.

Despite the positive outcomes throughout advanced preclinical and clinical evaluations of the anticancer ruthenium(III)-based compounds NAMI-A^15^ - the most advanced candidate drug having completed Phase II clinical trials - and KP1019^16^ (NKP1339, the sodium salt of KP1019, is described to be ready for clinical appliance^17^), various drawbacks have been observed, mainly related to their limited stability in physiological conditions, impairing both their general pharmacokinetic and pharmacodynamic profiles^18^. In addition to interfering with the internalization process into targeted cancer cells, degradation events can also limit the therapeutic effectiveness, thereby becoming a central issue that has claimed a reconsideration of the efficacy of low molecular weight ruthenium complexes currently in preclinical or clinical studies^19^. Additionally, the lack of convincing evidences on their biological mode of action, as well as on targets identification, has significantly limited their further development^20^,^21^. Aiming at improving the stability of the ruthenium(III)-based drugs in biological environments, as well as their suitability for biomedical applications, we have recently proposed an innovative strategy for their transport *in vivo* incorporating them within liposomial aggregates. Starting from a core molecular scaffold based on a ruthenium complex inspired to NAMI-A, named AziRu showing *per se* higher *in vitro* cytotoxicity than NAMI-A^22^,^23^, we have developed a mini-library of highly functionalized amphiphilic ruthenium(III) complexes, including differently decorated nucleolipids (see Fig. 1). The resulting nucleolipidic Ru(III) complexes (named ToThyRu, HoThyRu and DoHuRu) are endowed with a marked propensity for self-aggregation in aqueous solutions and high *in vitro* antiproliferative activity against human cancer cells, thus proving to be promising lead compounds for future *in vivo* studies. Moreover, on the lookout for biomimetic nanosystems, stable nucleolipidic Ru(III) complexes were produced by co-aggregation with either the zwitterionic phospholipid POPC (1-palmitoyl-2-oleoyl-*sn*-glycero-3-phosphocholine) or the cationic lipid DOTAP (1,2-dioleoyl-3-trimethylammoniumpropane chloride). The final nanoaggregates - which exhibit liposomial structure and have been subjected to an in-depth microstructural characterization - are *ad hoc* designed to present high stability under physiological conditions as well as to transport high ruthenium amounts in cells, thereby ensuring more effective metal-based treatments^24^,^25^,^26^,^27^. Indeed, recent *in vitro* bioactivity investigations have shown the great potential of our biocompatible ruthenium-containing liposomes as active antineoplastic agents against human carcinoma cells of different histological origin^24^. Of special interest have been the results obtained on estrogen and progesterone receptor positive MCF-7 adenocarcinoma cells, an *in vitro* model reflecting to a large extent the features of luminal breast cancer cells *in vivo*, worldwide the most common invasive cancer in women. Most remarkably, liposomes loaded with the Ru(III)-containing nucleolipids have shown a promising selectivity towards cancer cells in bioscreens *in vitro*^19^,^22^,^25^,^26^,^27^.

To expand knowledge on these compounds so to optimally develop ruthenium-based candidate drugs, mechanisms including interaction with DNA and induction of apoptosis have been investigated, but little has been hitherto explored on their overall modes of action as well as on biomolecular targeting^14^. Thus, in continuation with our previous studies, we have herein investigated in-depth the anticancer profile and mode of action of Ru(III)-containing liposomes. In particular, by focusing on well-established breast cancer cell lines as *in vitro* models for human mammary tumours, we here describe *via* preclinical bioscreens how nucleolipidic ruthenium complexes are able to activate specific molecular cell death pathways, thereby interfering with cancer cells growth and proliferation. We also provide evidence that could strengthen novel drug-discovery strategies and ultimately lead to more selective ruthenium-based anticancer agents.

## Results

### *In vitro* bioscreens for anticancer activity

The antiproliferative properties of the liposomes containing the Ru(III)-nucleolipids were screened on human breast cancer models by means of selected mammary malignant cells endowed with different phenotypic and/or genotypic features and different replicative and/or invasive potential (MCF-7, CG-5, MDA-MB-231, MDA-MB-468 and MDA-MB-436 cell lines). The concentration-effect curves (Fig. 2a) - here reported in terms of a “cell survival index” which combines the measurements of cell number and viability - show a typical concentration-dependent sigmoid trend yielding IC_50_ values in the low micromolar range. According to the IC_50_ values (see Table 1), the ToThyRu/DOTAP and DoHuRu/DOTAP formulations are the most effective in reducing the proliferation of all the used cell lines. This result, consistent with our previous reports, is likely associated to the positive charge of DOTAP formulations which can promote the interaction with the plasma membranes allowing a faster and quantitative drug cellular uptake^19^. Moreover, in relation to their effectiveness *in vitro*, there are no significant differences between the ToThyRu and DoHuRu nucleolipidic Ru(III) complexes used in our liposomial formulations, consistently with the rationale that the ruthenium center is the bioactive species, and the nucleolipids are simply carrier molecules^24^. IC_50_ data normalization in favour of the actual ruthenium content enclosed in ToThyRu/DOTAP and DoHuRu/DOTAP liposomes (15% mol/mol), leads up to values of IC_50_ of about 3–4 μM in CG-5 cells and of about 10 μM in MCF-7 cells, suggestive of a marked antiproliferative bioactivity *in vitro*. Overall, the calculated ruthenium IC_50_ values for DOTAP liposomes are in the low micromolar range (≤20 μM), similar or lower than those measured for cisplatin used in the same experimental conditions as a reference drug. All the breast cancer cells used in this screening are therefore sensitive to the action of metal-based drugs. The ruthenium IC_50_ values relative to cell growth inhibition in the presence of ToThyRu/POPC and DoHuRu/POPC formulations are also relevant, reaching values below 20 μM as in the case of MDA-MB-468, MCF-7 and CG-5 cancer cells. Looking further at IC_50_ values, the “naked” low molecular weight Ru(III) complex AziRu exhibits a milder antiproliferative activity on the same breast cancer cells, showing IC_50_ values constantly greater than 250 μM. These values are generally in agreement with those reported in the literature for the antimetastatic NAMI-A^28^, and once more highlight the critical importance of the delivery strategy to ensure the drug stability in the extra-cellular environment, a quantitative transport across the membranes and the bioavailability at the biological targets. Noteworthy, the bioactivity potentiating factors calculated as the ratio of IC_50_ values of the Ru(III)-containing liposomes with respect to the IC_50_ of AziRu, reach values of more than 20 in terms of antiproliferative ability^27^.

### Cellular morphological changes

Monitoring the cellular morphological changes over time can provide an additional support to the antiproliferative effects produced by ruthenium-based nanoaggregates. Throughout the *in vitro* trials, imaging end-points were collected by light microscopy from cells to assess cellular morphological changes induced by drug administration. Microphotographs in Fig. 2b of MCF-7 and MDA-MB-231 breast adenocarcinoma cells, nowadays among the most reliable *in vitro* models of breast cancer, in the presence of DoHuRu/POPC and DoHuRu/DOTAP (at their IC_50_ concentrations for 48 and 72 h) are suggestive of the characteristic cell shrinkage as well as loss of cell-cell contact that accompanies apoptosis occurrence. Moreover, after *in vitro* treatments cells were morphologically examined at a higher magnification by a phase-contrast microscope for autophagic vacuoles formation. As shown in Fig. 2c, in addition to apoptotic hallmarks, autophagic vacuoles clearly appear when MCF-7 cells were treated with the DoHuRu/DOTAP liposomes for 48 h at IC_50_ concentration, providing a morphological support of autophagy activation. These observations were further confirmed by FACS analysis, discussed below. Thus, the AziRu *in vitro* treatment by the DoHuRu/POPC liposome seems to trigger exclusively apoptosis, whereas the one with the cationic DoHuRu/DOTAP seems to simultaneously activate apoptosis and autophagy.

### Pro-apoptotic effects in breast cancer cells

Changes both in the cell morphology and in the monolayers appearance suggest that liposomes containing the nucleolipidic Ru(III)-complexes might have chemotherapeutic effects in human breast cancer initiating cells towards specific cell death pathways. The evaluation of apoptosis induction *via* FACS analysis has revealed that both DoHuRu/POPC and DoHuRu/DOTAP formulations are able to induce remarkable pro-apoptotic effects on MCF-7 cells and MDA-MB-231. In fact, as depicted in Fig. 3a,c with reference to MCF-7 cells, the 46% of total cell population is in advanced stage of apoptosis following 48 h of exposure to IC_50_ concentrations of DoHuRu/DOTAP; further 24 h of treatment results in the 82% of cell population in late apoptosis phase. Milder pro-apoptotic effects, but nonetheless of significance, are detected in the case of DoHuRu/POPC treatment, leading after 48 h to about 36% of total MCF-7 cell population in early apoptosis and to a 37% in late apoptosis after 72 h. As a further validation of these data, no significant signals of apoptosis perturbation, such as an increased necrosis, were detectable by FACS analysis. A similar distribution of cell population among the different apoptotic stages has been observed by investigating the effects of liposomes containing nucleolipidic Ru(III)-complexes on MDA-MB-231 cells (see Fig. 3b,d), confirming the increased *in vitro* efficacy - especially as a trend over time - of the cationic nanoaggregates (*i.e.*, the DoHuRu/DOTAP ones) with respect to the zwitterionic POPC ones. This scenario is fully consistent with our previous studies^19^,^27^. As a result of different cellular uptake kinetics, ruthenium-containing cationic liposomes (DoHuRu/DOTAP) induce faster biological effects by way of cellular apoptosis activation than those dependent by the neutral liposomes (DoHuRu/POPC).

### DNA fragmentation in MCF-7 and MDA-MB-231

It is generally accepted that DNA damage and subsequent induction of apoptosis is the primary cytotoxic mode of action of cisplatin and other metal-based antiproliferative drugs. In addition to shrinkage and fragmentation of the cells and nuclei, the apoptotic process is accompanied by degradation of the chromosomal DNA into nucleosomal units. Indeed, late events of apoptosis typically lead to DNA cleavage, resulting in a “ladder” formation detectable by agarose gel electrophoresis. To verify apoptosis induction in breast cancer cells in a direct manner, we analyzed DNA degradation on genomic DNA samples obtained from treated cells. As depicted in Fig. 3e, though with a non-canonical nuclear fragmentation pattern in MCF-7 due to inherent caspase-3 expression deficiency, the DNA damage markedly increased in cells exposed to both DoHuRu/POPC and DoHuRu/DOTAP for 48 h at IC_50_ doses. Moreover, the typical internucleosomal DNA laddering of cell undergoing apoptosis clearly appeared in MDA-MB-231 cells, with a fragmentation pattern similar to that induced by IC_50_ doses of cisplatin *in vitro*, herein used as positive control^29^.

### Apoptotic-related proteins in MCF-7 and MDA-MB-231 breast cancer cells

Given that the expression profile of pro-apoptotic proteins is a central determinant for the death mode induced by ruthenium-based drugs, we analyzed the expression of proteins linked to the two main pathways that control mammalian apoptosis, *i.e.* the extrinsic (or death receptor) and intrinsic (or mitochondrial) apoptotic pathways. As determined by Western blot analysis reported in Fig. 4a,c,e, protein extracts from MCF-7 exposed for 48 and 72 h to IC_50_ concentrations of DoHuRu/POPC and DoHuRu/DOTAP showed a remarkable increase in caspase-9 activity compared with control, whereas in the same experimental conditions no caspase-8 activation was detectable. Indeed, the activation of pro-caspase-9 involves intrinsic proteolytic processing, resulting in the production of an active p35 subunit. Moreover, as clearly detectable by immunoblot, an additional cleavage occurred producing a large p37 subunit which is known to amplify the apoptotic response. On the other hand, full length pro-caspase-8 expression was not affected by *in vitro* treatments. Taken together, these results suggest a ruthenium-dependent activation of the apoptotic mitochondrial pathway, wherein Bax and Bid are key cell death factors increasing mitochondrial permeability and release of cytochrome *c*. Conversely, cell survival factor Bcl-2 inhibits actions of Bax and Bid on mitochondria^30^. In MCF-7 cells Bax was significantly increased and Bcl-2 decreased upon DoHuRu/POPC and DoHuRu/DOTAP treatment - a cellular response predisposing to apoptotis activation. These biological effects were time-dependent and more evident following *in vitro* treatment with the cationic DoHuRu/DOTAP liposomes.

In the same way, ruthenium treatment in MDA-MB-231 breast cancer cells *via* DoHuRu/POPC and DoHuRu/DOTAP *in vitro* administration at IC_50_ concentrations elicited caspase-9 activation after 48 h, and this anew was coupled to the simultaneous Bax and Bcl-2 up-regulation and down-regulation, respectively (Fig. 4b,d,f). Furthermore, in this breast cancer cell line DoHuRu/DOTAP seemed able to promote full length pro-caspase-8 cleavage, as evidenced by the formation of the active p10 and p18 fragments. In turn, activated initiator caspases further process other caspase members, including caspase-3 and caspase-7, to initiate a caspase cascade, which generally leads to complete the programmed cell death process. In fact, the immunoblotting analysis performed on MDA-MB-231 cells exposed to Ru(III)-containing liposomes further revealed a marked proteolytic cleavage of the inactive proenzyme to activate caspase-3. Hence, the evaluation of apoptosis regulatory proteins in breast cancer models for the *in vitro* preclinical evaluation of ruthenium biological effects suggests the invariable induction of the mitochondrial apoptotic cell death pathway, but also the cell-specific activation of the extrinsic death pathway particularly in the case of the cationic nanoaggregate.

Interestingly, the treatment with either DoHuRu/POPC or DoHuRu/DOTAP alters the expression profile of Bax and Bcl-2 proteins with respect to basal amounts, radically changing the Bax/Bcl-2 ratio. The Bax up-regulation and Bcl-2 down-regulation observed in concert following ruthenium action may predispose cells to apoptosis^30^.

### Autophagy activation in MCF-7 and MDA-MB-231 breast cancer cells

In addition to apoptosis, cellular suicide can also be executed *via* non-apoptotic forms of programmed death such as autophagy. The simultaneous evaluation of apoptosis and autophagy by means of the herein used methods is not mutually exclusive, *i.e.* it is possible to ascertain the degree of autophagy independently from that of apoptosis. Thus, to investigate whether DoHuRu/POPC and DoHuRu/DOTAP can also induce autophagy in MCF-7 and MDA-MB-231 cells, we have examined the formation of autophagic vacuoles using monodansylcadaverine (MDC), a selective autofluorescent dye for autolysosomes detection. Autolysosomes occurrence, which results from lysosomes-autophagosomes fusion, exclusively characterizes late steps in the autophagic cell death process. In order to verify this circumstance, cells were exposed for 48 and 72 h to ruthenium IC_50_ concentrations enclosed within DoHuRu/POPC and DoHuRu/DOTAP nanoaggregates (18.9 and 10.3 μM, respectively for MCF-7, 14.7 and 12.1 μM, respectively for MDA-MB-231) and the quantitative evaluation of MDC staining by means of FACS analysis was performed (see Fig. 5). As a result of MDC accumulation and consistently with phase contrast cell imaging, the cationic DoHuRu/DOTAP formulation induced an evident increase in the Mean Fluorescence Intensity (MFI), in particular 4.3 and 5.8-fold higher at 48 and 72 h for MCF-7, and 3.9 and 5.3-fold higher for MDA-MB-231 than untreated control cells. These results indicate an increased formation of the MDC-labeled vacuoles after ruthenium treatment *via* DoHuRu/DOTAP cationic liposomes and suggest the activation of autophagy in presence of apoptosis. Conversely, by using the zwitterionic DoHuRu/POPC liposomes, no significant autolysosomes formation was detected, at least up to 72 h.

## Discussion

Innovative anticancer drugs with new molecular mechanisms of action are essential in chemotherapeutic treatment to kill specific cancer types, and to overcome toxic side effects as well as chemoresistance^31^. Current research efforts are focused on a deeper understanding of the cellular response and/or resistance to anticancer treatments, including the role of cell death pathways activation, such as apoptosis and autophagy, by chemotherapeutics^32^. Recently, we have developed new biocompatible ruthenium-based nanosystems, proved to be particularly effective against specific cell lines derived from human solid tumours^19^,^22^,^24^,^25^,^26^,^27^. Starting from these encouraging data, in the present work we have first confirmed their efficacy focusing on a panel of human tumour cells arising from breast cancer. At the moment, the endocrine-responsive (ER) breast adenocarcinoma MCF-7 and the triple-negative breast adenocarcinoma (TNBC) MDA-MB-231 cell models account for the great majority of investigations on breast cancer cells and are considered the most reliable *in vitro* models of breast cancer together with their variants CG5, and MDA-MB-436 and MDA-MB-468, respectively^33^,^34^. All these cells are sensitive to cisplatin *in vitro*, so that cisplatin is used in standard first line treatment protocols for many breast cancers, often in combination with other drugs, such as taxanes, vinca alkaloids, and 5-fluorouracil, resulting in synergistic or additive effects. Therefore, classical chemotherapy is currently the only drug-based option in the therapeutic armamentarium, and metal-based chemotherapy is also emerging as an upcoming treatment modality especially in TNBC^35^. Although new types of ruthenium complexes with superior anticancer activity *in vitro* have been meanwhile reported^36^, the induction of comparable or even greater cytotoxic effects than cisplatin have been herein confirmed for nucleolipidic Ru(III) formulations. Then, aiming at an in-depth investigation of their mode of action, we have shown that amphiphilic ruthenium complexes, properly delivered by suitable nano-systems, are able to kill cancer cells by activating specific apoptotic processes, coupled in some cases to cellular autophagy. Indeed, in the last decade many different ruthenium compounds have been tested for their anticancer properties. However, all the reported studies have not clearly evidenced a unique mode of action at cellular level, nor unambiguously defined a specific mechanism of action at molecular level^10^,^21^,^24^. In-depth bioanalytical studies have been so far performed especially for cisplatin, which have validated nuclear DNA as the main final drug target causing adducts formation and DNA damage, leading to cell division inhibition and cytotoxicity^5^. In analogy with cisplatin, some of us have recently demonstrated that also AziRu and NAMI-A can interact with DNA model systems, with Ru(III) ions being incorporated into oligonucleotide structures *via* stable linkages^37^. However, since an increasing number of evidences demonstrate that both ruthenium(II) and (III)-based drugs are able to interact with both intra- and extra-cellular protein targets, currently other molecular mechanisms of action cannot be excluded^9^,^38^.

Beyond molecular targeting, we have observed an invariable activation of programmed cell death pathways which confirms that the primary mode of action in breast cancer models of AziRu *via* DoHuRu/POPC and DoHuRu/DOTAP formulations is the induction of apoptosis. These data are largely in accordance with several reports highlighting the occurrence of distinct hallmarks of apoptosis after ruthenium administration *in vitro*^16^,^39^. Cellular morphological changes and DNA fragmentation provided additional evidence of an apoptosis-inducing activity at the basis of the anticancer properties of AziRu. The regulation of apoptotic cell death orchestrated by intracellular caspases plays a fundamental role in the response to chemotherapeutics, as evasion of apoptosis is one of the central features of malignant progression as well as of drug resistance. Indeed, survival of malignant mammary cells is a key event in disease occurrence and progression, but also in therapy failure and chemoresistance development^40^. Physiological mammary cells growth is controlled by a balance between cell proliferation and apoptosis. A large body of evidence has clarified that tumour growth is not just a result of uncontrolled proliferation but also of reduced apoptosis, so that the balance between proliferation and apoptosis is crucial in determining the overall growth or regression of the tumour in response to chemotherapy^41^. Two main pathways of caspase activation have been described in mammalian cells, which result in final control of apoptosis. In the intrinsic pathway, typically activated by intracellular stress signals, pro-apoptotic cell death factors belonging to the Bcl-2 family increase mitochondrial permeability and release of cytochrome *c*, as well as of other proteins from the intermembrane space of mitochondria. Apaf-1, a downstream mediator of apoptosis, along with cytochrome *c*, associates with caspase-9 in cytoplasm and leads to its activation^42^. The resulting apoptosome initiates a cascade of effector caspases, which include caspases-3, -6, and -7. In turn, the active caspase-3 triggers DNA fragmentation factor (Caspase-Activated DNase, CAD) and promotes DNA internucleosomal cleavage. All the formulations containing nucleolipidic Ru(III)-complexes we have tested activate the mitochondrial apoptotic cell death pathway in breast cancer cells, as highlighted by a remarkable activation of caspase-9. Interestingly, this occurs independently of the cell ability to complete apoptosis process *via* the executioner caspase-3, as demonstrated by Ru-dependent activation of apoptosis in MCF-7. This adenocarcinoma model is known to be resistant to some chemotherapeutics due to a deletion in the CASP-3 gene that leads to an inherited deficiency of caspase-3^43^. Caspase-3 - commonly turned on by numerous death signals - cleaves a variety of important cellular proteins and is ultimately responsible for apoptotic DNA fragmentation. Despite the lack of caspase-3 expression, liposomes containing nucleolipidic Ru(III)-complexes have hitherto shown to be particularly effective on this *in vitro* model. It has been also reported that MCF-7 undergoes cell death by apoptotic stimuli in the absence of the typical DNA fragmentation, and recent observations further suggest that large and small DNA fragments coupled to even single-strand cleavage events occur during apoptotic death^44^. Consistently with our results, these observations have raised relevant questions on the degradation pattern of nuclear DNA in agarose gel electrophoresis detection, which remains controversial. However, morphological changes and MCF-7 cell death were independent of caspase-3 and may correlate with the activation of different apoptotic pathways and other effector caspases, such as caspase-6 or -7. As far as the activation of programmed cell death pathways is concerned, DoHuRu/DOTAP formulation seems capable to concurrently activate the two major pathways of apoptosis in a cell-specific mode. In fact, MDA-MB-231 cells, undergoing the apoptotic mitochondrial pathway after exposure to DoHuRu/DOTAP, show an apparent proteolytic processing of pro-caspase-8 to form various fragments, including the active p18 and p10. The extrinsic pathway is activated by extracellular ligands able to bind to death receptors on the cell surface, which leads to the formation of the death-inducing signaling complex (DISC). This death receptor pathway is triggered by members of the death receptor superfamily such as CD95 and tumour necrosis factor receptor. Formation of a death-inducing signalling complex induces caspase-8 activation and thereby the downstream caspase cascade^45^. We hypothesize that the cationic DOTAP nanoaggregate, by means of its inherent surface charge, can interact in peculiar manner with the external surface of cell membranes, as suggested by the faster cellular uptake kinetics compared to POPC formulations observed in a previous study^19^. Although a hypothesis, exclusive molecular interactions coupled to local drug release could stimulate specific surface receptors involved in the activation of the extrinsic pathway. Indeed, not infrequently the killing of tumor cells by anticancer chemotherapeutics has been linked to activation of extrinsic apoptosis pathways^46^. These outcomes are consistent with former investigations and demonstrate that some ruthenium(II) and (III) complexes can simultaneously trigger intrinsic and extrinsic apoptosis pathways^47^.

As far as the Bcl-2 family proteins are concerned, we believe that some central aspects closely linked to ruthenotherapy have emerged. Members of this family are regulatory proteins involved in the control of cell death, by either inducing (pro-apoptotic factors) or inhibiting (anti-apoptotic factors) apoptosis^48^. Bcl-2 is a crucial anti-apoptotic protein to be regarded as an oncogene, playing an important role in promoting cellular survival by inhibition of pro-apoptotic proteins. On the other hand, the pro-apoptotic effectors of the Bcl-2 family, including Bax, normally act on the mitochondrial membrane to promote permeabilization coupled to the release of both cytochrome *c* and ROS as important signals in the apoptosis cascade. Several clinical studies have provided support for an overexpression of the antiapoptotic Bcl-2 protein as a negative prognostic marker in various tumours^49^. Alternatively, decreased Bax levels have been found in correlation with shorter survival in patients with breast cancer and colorectal cancer^50^. In MCF-7 and MDA-MB-231 we found an important effect induced by both DoHuRu/POPC and DoHuRu/DOTAP treatment on the cellular content of Bcl-2 and Bax, which could be correlated to the induction of the mitochondrial cell death pathway. The significant increase in Bax/Bcl-2 ratio detected in treated cells with respect to untreated cells could have an important impact in the regulation of cell fate by interfering with breast cancer cell survival. Indeed, in accordance with experimental and clinical investigations, tumours dependent on Bcl-2 family members are likely sensitive to Bcl-2 modulation in order to survive; in turn high Bax expression has been associated with a better response to chemotherapy in many cancers forms^51^. Consistently with our results, it should be possible to circumvent the inherent apoptosis deficiency of malignant cells by directly affecting the mitochondrial function. In this way, following mechanisms yet to be clarified, the ruthenium complexes may interact with mitochondrial targets, possibly *via* selective accumulation and ROS generation^52^. At the same time, dysfunction of mitochondria might be responsible for autophagic cell death. More and more studies underline the important interplay between apoptosis and autophagy, and suggest that apoptosis activation is often related to increased autophagy processes^53^. As in the case of apoptosis, autophagy plays a vital role in cellular proliferation and survival, and dysregulated autophagy activation has been described in many pathologies, including cancer. Although the role of the autophagic programmed cell death in neoplastic development remains to be clarified *in vivo*, cancer cells with up-regulated autophagy exhibit a less aggressive behaviour as well as an increased susceptibility to chemotherapy^54^. Since the effectiveness of many apoptosis-inducing chemotherapies can be affected by specific mutations in genes orchestrating apoptotic regulation, the activation of alternative cell death pathways, in tandem with or in the absence of an efficient apoptotic machinery, may represent an attractive goal for novel metal-based chemotherapeutics. In this perspective, recent evidence supports the occurrence of the simultaneous induction of apoptosis and autophagy in cancer cells as a result of specific signaling^55^. In addition, autophagy can be also activated upon exposure to genotoxic compounds, including several metal-based drugs able to target DNA. The induction of autophagy in the case of cationic nucleolipidic formulations could be linked to the ruthenium-induced down-regulation of the prosurvival protein Bcl-2, evident in both MCF-7 and MDA-MD-231 cells. Indeed, disturbances in the interaction between Beclin-1 and Bcl-2 family proteins, by which Beclin-1 is inhibited in normal conditions, has been established to stimulate cellular autophagy^56^. Thus Ru(III) complexes activity in inhibiting breast cancer cells proliferation *via* DoHuRu/DOTAP administration would lie in the crosstalk connecting the main molecular mechanisms involved in the regulation of apoptosis and autophagy processes. Nevertheless, the existence of other non-apoptotic nor necrotic cell death pathways, triggered upon exposure to ruthenium-containing nanoaggregates we have documented in breast cancer models, needs further and more targeted studies. According to our previous findings^24^ and to recent literature reports^36^,^52^, it cannot be excluded that the simultaneous activation of different mechanisms of cell death can be caused by multiple potential interactions at the subcellular/molecular level, both nuclear and cytosolic. Nevertheless, the activation of multiple death pathways by metal-based chemotherapeutics in aggressive cancer diseases with limited treatment options is a largely desired goal, in order to possibly restrict the onset of chemoresistance as well as to efficiently counteract uncontrolled proliferation. Upcoming investigations on the critical proteins and pathways involved in autophagy control exerted by Ru(III)-containing liposomes are underway to strengthen knowledge in favour of future *in vivo* applications for these high potential candidate drugs.

## Methods

### Cell cultures

Human breast adenocarcinoma cells, epithelial-like type as MCF-7, CG-5, MDA-MB-231 and MDA-MB-468 lines, and pleomorphic-like type as MDA-MB-436 line, were grown in DMEM (Invitrogen, Paisley, UK) supplemented with 10% fetal bovine serum (FBS, Cambrex, Verviers, Belgium), L-glutamine (2 mM, Sigma, Milan, Italy), penicillin (100 units/ml, Sigma) and streptomycin (100 μg/ml, Sigma), and cultured in a humidified 5% carbon dioxide atmosphere at 37 °C, according to ATCC recommendations. MCF-7 and CG-5 are endocrine-responsive (ER) breast cancers; MDA-MB-231, MDA-MB-468 and MDA-MB-436 are triple-negative breast cancers (TNBC)^33^,^34^. Human HaCaT keratinocytes and rat L6 skeletal muscle cells, used as healthy control cell lines, were grown in the same experimental conditions.

### Synthesis of the ruthenium complexes ToThyRu and DoHuRu and preparation of the lipid-based nanoaggregates

**Figure 1:**
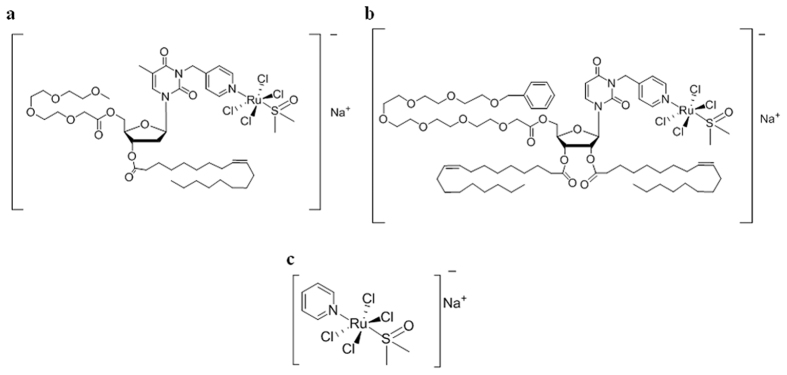
Molecular structures of Ru(III)-containing complexes. The investigated Ru(III) nucleolipidic complexes (**a**) ToThyRu and (**b**) DoHuRu, along with the low molecular weight Ru(III) complex (**c**) AziRu.

**Figure 2:**
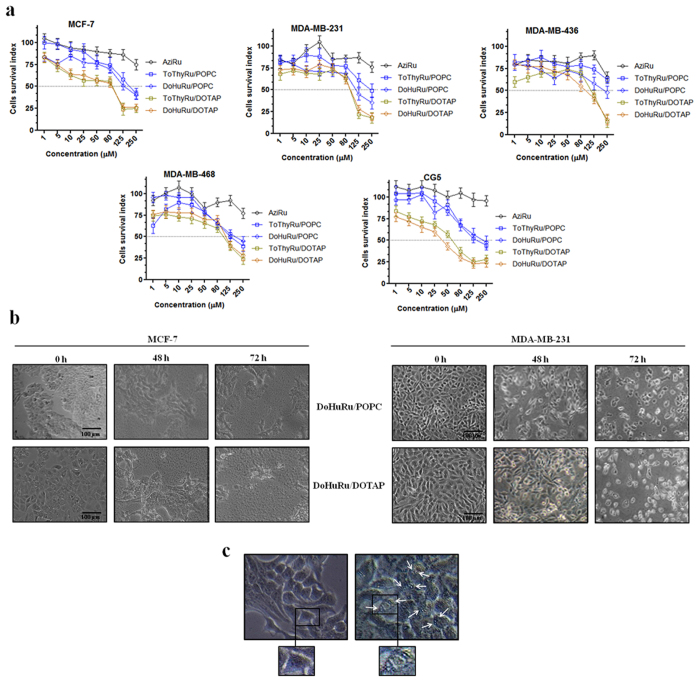
Cell survival index and cytomorphological alterations on cell monolayers. **(a)** Cell survival index, evaluated by the MTT assay and monitoring of live/dead cell ratio for MCF-7, MDA-MB-231, MDA-MB-436, MDA-MB-468, and CG-5 cell lines following 48 h of incubation with the indicated concentration (the range 1 → 1000 μM has been explored, the one 1 → 250 μM is shown) of AziRu, and of the ruthenium-containing (15% mol/mol) POPC formulations (ToThyRu/POPC and DoHuRu/POPC) and DOTAP formulations (ToThyRu/DOTAP and DoHuRu/DOTAP), as indicated in the legend. Data are expressed as percentage of untreated control cells and are reported as mean of five independent experiments ± SEM (*n* = 30). **(b)** Representative microphotographs at a 200 × magnification (20 × objective and a 10 × eyepiece) by phase-contrast light microscopy of MCF-7 (left panels) and MDA-MB-231 (right panels) breast cancer cells treated for 48 and 72 h with ruthenium IC_50_ micromolar concentrations of DoHuRu/POPC (18.9 and 14.7 μM, respectively) and DoHuRu/DOTAP liposomes (10.3 and 12.1 μM, respectively), showing the cellular morphological changes and the cytotoxic effects on cell monolayers. The shown images are representative of three independent experiments. **(c)** Representative microphotographs of untreated (left panel) and 48 h DoHuRu/DOTAP treated (right panel) MCF-7 cells by phase-contrast light microscopy at a 600 × magnification (30 × objective and a 20 × eyepiece). DoHuRu/DOTAP (at IC_50_ concentration) induces the formation of autophagic vacuoles detectable in cell cytoplasm. Inset: higher magnifications of MCF-7 cells before and after treatment.

**Figure 3:**
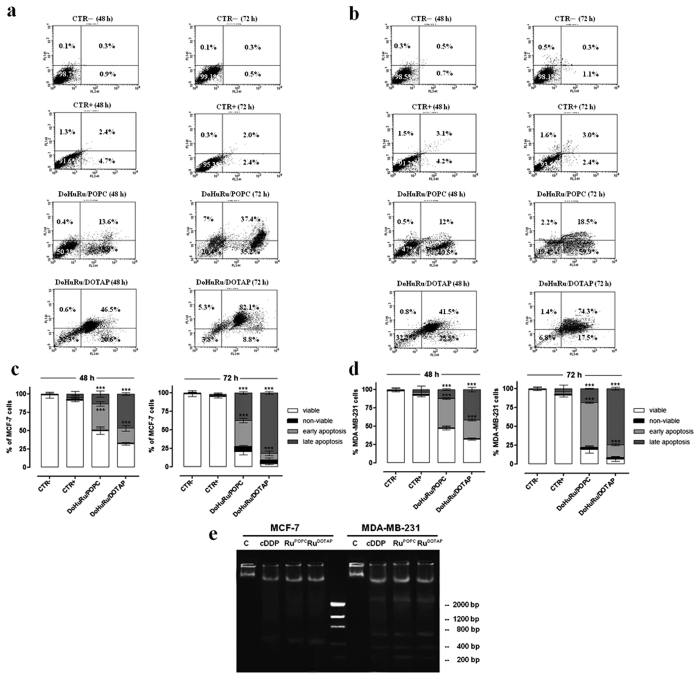
Induction of apoptosis in MCF-7 and MDA-MB-231 breast cancer cells. Apoptosis was evaluated by FACS analysis, after cell labeling with propidium iodide (PI) and FITC-Annexin V. MCF-7 **(a)** and MDA-MB-231 **(b)** cells were both unlabeled and untreated (CTR−), labeled and not treated (CTR + ), treated with DoHuRu/POPC or with DoHuRu/DOTAP for 48 and 72 h using IC_50_ concentrations, as indicated. The lower left quadrants of each panels show the viable cells, which exclude PI and are negative for FITC-Annexin V binding. The upper left quadrants contain the non-viable, necrotic cells, negative for FITC-Annexin V binding and positive for PI uptake. The lower right quadrants represent the cells in early apoptosis, that are FITC-Annexin V positive and PI negative. The upper right quadrants represent the cells in late apoptosis, positive for both FITC-Annexin V binding and for PI uptake. The experiments were performed at least three times with similar results. Quantitative analysis of viable, non-viable (necrotic), early and late apoptotic MCF-7 **(c)** and MDA-MB-231 **(d)** cells after 48 and 72 h of treatments are shown. Data are expressed as percentage of untreated control cells and are reported as mean of four independent experiments ± SEM (*n* = 24); ***p < 0.001 *vs.* control (untreated cells). **(e)** DNA fragmentation assay on MCF-7 and MDA-MB-321 cells treated or not (C) with IC_50_ concentrations of DoHuRu/POPC (Ru^POPC^) and DoHuRu/DOTAP (Ru^DOTAP^) for 48 h, and with IC_50_ doses (17 and 19 μM, respectively) of cisplatin (*c*DDP) as the positive control for DNA fragmentation. After incubation, the DNA was extracted and visualized on 1.5% agarose gel as detailed in the Methods section. The lane in the middle corresponds to the molecular weight markers. The agarose gel is representative of three independent experiments.

**Figure 4:**
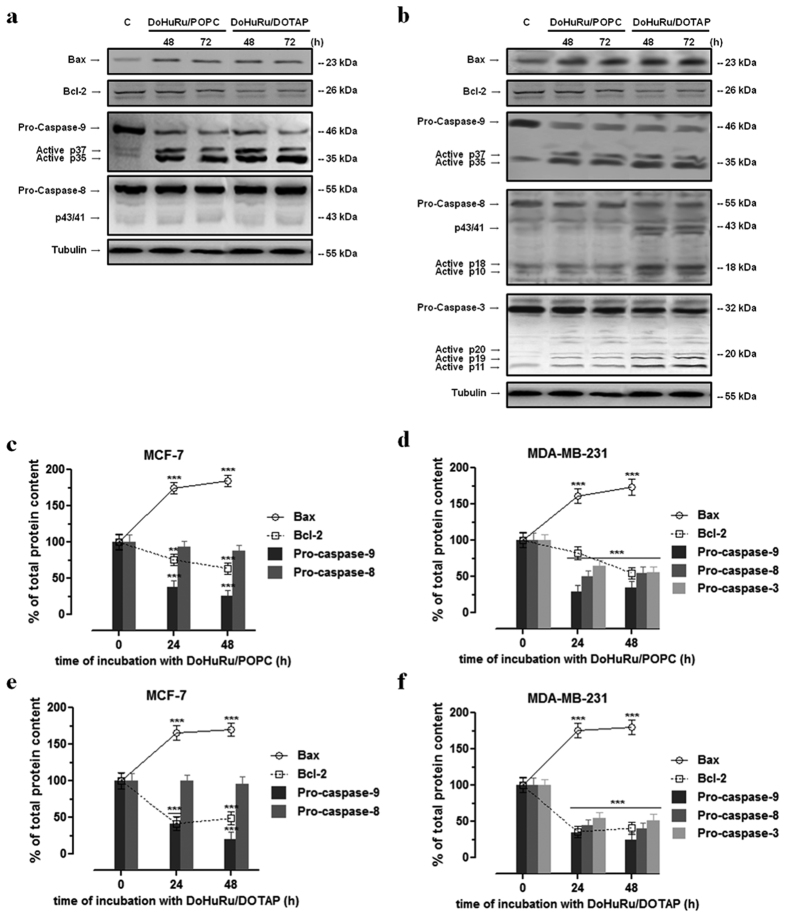
Apoptotic regulatory proteins in MCF-7 and MDA-MB-231 cells. Western blot analysis showing the effects of IC_50_ concentrations of DoHuRu/POPC and DoHuRu/DOTAP following 48 and 72 h of incubations in MCF-7 **(a)** and MDA-MB-231 **(b)** cells on caspases-3, -8 and -9 expression and activation, and on Bax and Bcl-2 expression, to characterize the apoptotic response. Shown are blots representative of four independent experiments. After chemoluminescence, the bands resulting from MCF-7 **(c,e)** and MDA-MB-231 **(d,f)** cell extracts were quantified by densitometric analysis and plotted in line (solid and dotted line for Bax and Bcl-2 proteins, respectively) and bar (caspases-3, -8 and -9) graphs as percentage of control in relation to the used ruthenium-containing nanoaggregate, as indicated. Shown are the average ± SEM values of four independent experiments. The anti-tubulin antibody was used to standardize the amounts of proteins in each lane. **p < 0.01 *vs.* control cells; ***p < 0.001 *vs.* control cells.

**Figure 5:**
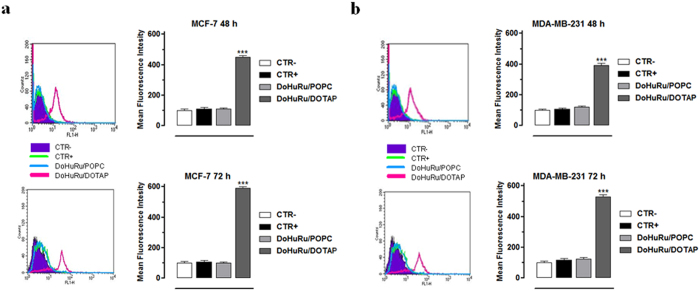
Autophagy activation in MCF-7 and MDA-MD-231 breast cancer cells. Quantitative flow cytometric analysis of autophagosomes formation (MDC incorporation) in MCF-7 **(a)** and MDA-MD-231 **(b)** breast cancer cells, unlabeled and untreated (CTR−), labeled and untreated (CTR+), treated with IC_50_ concentrations of DoHuRu/POPC or with DoHuRu/DOTAP for 48 and 72 h, as indicated. The main fluorescence intensities (MFIs) were calculated, as described in ‘Materials and Methods’. In the corresponding bar graphs, values are expressed as percentage of control cells and are reported as mean of four independent experiments ± SEM (*n* = 24); ***p < 0.001 *vs.* control (untreated cells).

The ruthenium complexes investigated, namely ToThyRu and DoHuRu (here depicted in Fig. 1 along with AziRu) were prepared by reacting in stoichiometric amounts the starting nucleolipids, named ToThy or DoHu^57^, with the Ru complex [*trans*-RuCl_4_(DMSO)_2_]^−^Na^+^ following a previously described procedure^25^. In all cases, the desired nucleolipidic Ru(III) complexes were obtained in a pure form, as confirmed by TLC and ESI-MS analysis, and almost quantitative yields. The lipid formulations of ToThyRu and DoHuRu in POPC and DOTAP were prepared as previously reported^24^ and characterized by DLS analysis.

### *In vitro* bioscreens

The anticancer activity of ruthenium-containing nucleolipidic nanoparticles and of AziRu was investigated through the estimation of a “cell survival index”, arising from the combination of cell viability evaluation with cell counting^25^. Cells were inoculated in 96-microwell culture plates at a density of 10^4^ cells/well, and allowed to grow for 24 h. The medium was then replaced with fresh medium and cells were treated for further 48 h with a range of concentrations (1 → 1000 μM) of AziRu, and of ToThyRu and DoHuRu complexes lodged either in POPC or DOTAP liposomes (ToThyRu/POPC and ToThyRu/DOTAP, DoHuRu/POPC and DoHuRu/DOTAP, respectively). Using the same experimental procedure, cell cultures were also incubated with ruthenium-free ToThy/POPC, ToThy/DOTAP, DoHu/POPC and DoHu/DOTAP liposomes as negative controls, as well as with cisplatin (*c*DDP) - a positive control for cytotoxic effects^27^. Cell viability was evaluated using the MTT assay procedure, which measures the level of mitochondrial dehydrogenase activity using the yellow 3-(4,5-dimethyl-2-thiazolyl)-2,5-diphenyl-2H-tetrazolium bromide (MTT, Sigma) as substrate. The assay is based on the redox ability of living mitochondria to convert dissolved MTT into insoluble purple formazan. Briefly, after the treatments, the medium was removed and the cells were incubated with 20 μl/well of a MTT solution (5 mg/mL) for 1 h in a humidified 5% CO_2_ incubator at 37 °C. The incubation was stopped by removing the MTT solution and by adding 100 μl/well of DMSO to solubilize the obtained formazan. Finally, the absorbance was monitored at 550 nm using a microplate reader (iMark microplate reader, Bio-Rad, Milan, Italy). Cell number was determined by TC10 automated cell counter (Bio-Rad, Milan, Italy), providing an accurate and reproducible total count of cells and a live/dead ratio in one step by a specific dye (trypan blue) exclusion assay. Bio-Rad’s TC10 automated cell counter uses disposable slides, TC10 trypan blue dye (0.4% trypan blue dye w/v in 0.81% sodium chloride and 0.06% potassium phosphate dibasic solution) and a CCD camera to count cells based on the analyses of captured images. Once the loaded slide is inserted into the slide port, the TC10 automatically focuses on the cells, detects the presence of trypan blue dye and provides the count. When cells are damaged or dead, trypan blue can enter the cell allowing living cells to be counted. Operationally, after treatments in 96-microwell culture plates, the medium was removed and the cells were collected. Ten microliters of cell suspension, mixed with 0.4% trypan blue solution at 1:1 ratio, were loaded into the chambers of disposable slides. The results are expressed in terms of total cell count (number of cells per ml). If trypan blue is detected, the instrument also accounts for the dilution and shows live cell count and percent viability. Total counts and live/dead ratio from random samples for each cell line were subjected to comparisons with manual hemocytometers in control experiments.

The calculation of the concentration required to inhibit the net increase in the cell number and viability by 50% (IC_50_) is based on plots of data (*n* = 6 for each experiment) and repeated five times (total *n* = 30). IC_50_ values were obtained by means of a concentration response curve by nonlinear regression using a curve fitting program, GraphPad Prism 5.0, and are expressed as mean values ± SEM (*n* = 30) of five independent experiments.

### Cell morphology

Human breast cancer cell lines were grown on 60 mm culture dishes by plating 5 × 10^5^ cells. After reaching the subconfluence, cells were incubated for 48 h with IC_50_ concentrations of the ruthenium-containing liposomes (DoHuRu/POPC and DoHuRu/DOTAP) under the same *in vitro* experimental conditions described above and were then morphologically examined by a phase-contrast microscope (Labovert microscope, Leizt) for autophagic vacuoles and apoptotic markers. Microphotographs at a 200 × total magnification (20 × objective and 10 × eyepiece) were taken with a standard VCR camera (Nikon).

### DNA fragmentation assay

MCF-7 and MDA-MB-231 cells were grown on standard plastic 60 mm culture dishes by plating 5 × 10^5^ cells. After 24 h of growth the cells were treated for 48 h with IC_50_ doses of DoHuRu/POPC and DoHuRu/DOTAP liposomes under the same experimental conditions described for bioscreens, as well as with IC_50_ doses of cisplatin (*c*DDP) - a positive control for *in vitro* apoptosis^29^. The DNA fragmentation assay was carried out as previously reported^27^. After treatments, cells were collected and the pellets were suspended in lysis buffer (50 mM Tris-HCl, pH 8.0, 5 mM EDTA, 100 mM NaCl, 1% SDS, 0.5 mg/mL Proteinase K) and incubated at 50 °C. After 1 h incubation, 10 mg/mL RNase was added to the lysates and incubated for 1 h at 50 °C. DNA was precipitated with NaOAc pH 5.2 and ice cold 100% EtOH and centrifuged at 14000 × *g* for 10 min. Pellets were dissolved in TE buffer (10 mM Tris-HCl, pH 8.0, 1 mM EDTA). A 20 μL aliquot of each DNA sample was analyzed on a 1.5% agarose gel stained with ethidium bromide and visualized under UV light.

### **F**ACS analysis

Annexin V-FITC (fluorescein isothiocyanate) was used in conjunction with a vital dye, propidium iodide (PI), to differentiate apoptotic (Annexin V-FITC positive, PI negative) from necrotic (Annexin V-FITC positive, propidium iodide positive) cells^58^. Briefly, cells were incubated with Annexin V–FITC (MedSystems Diagnostics, Vienna, Austria) and propidium iodide (Sigma, St. Louis, MO, USA) in a binding buffer (10 mM HEPES, pH 7.4, 150 mM NaCl, 5 mM KCl, 1 mM MgCl_2_, 2.5 mM CaCl_2_) for 10 min at room temperature, washed and resuspended in the same buffer. Analysis of apoptotic cells was performed by flow cytometry (FACScan, Becton 4 Dickinson)^59^. For each sample, 2 × 10^4^ events were acquired. The study was carried out by triplicate determination on at least three separate experiments.

### Labeling of autophagic vacuoles with monodansylcadaverine (MDC)

To quantify the induction of the autophagic process, MCF-7 and MDA-MB-231 cells, treated with IC_50_ concentrations of the DoHuRu/POPC and DoHuRu/DOTAP formulations, were stained with the autofluorescent agent monodansylcadaverine (MDC), a selective marker for autophagic vacuoles (AVOs), especially for autolysosomes^60^. Treated cells were incubated with 50 μM MDC in PBS at 37 °C for 15 min. After incubation, cells were washed twice with PBS, and immediately analyzed by flow cytometry with a FACScalibur flow cytometer (Becton Dickinson). The fluorescent emissions were collected through a 530 nm band pass filter (FL1 channel). At least 10^4^ events were acquired in log mode. For the quantitative evaluation of MDC, CellQuest software (Becton Dickinson) was used to calculate mean fluorescence intensities (MFIs). The MFIs were calculated by the formula (MFI treated/MFI control), where MFI treated is the fluorescence intensity of cells treated with the various compounds and MFI control is the fluorescence intensity of untreated and unstained cells. Values reported in the figures are the mean values ± SEM from three independent experiments.

### Preparation of cellular extracts

MCF-7 and MDA-MB-231 cells were cultured in standard plastic 60 mm culture dishes by plating 5 × 10^5^ cells. Behind reaching the subconfluence, cells were incubated for 48 and 72 h with IC_50_ concentrations of the ruthenium-containing liposomes (DoHuRu/POPC and DoHuRu/DOTAP) under the same experimental conditions herein described. After treatments, cells were washed and collected by scraping with PBS containing 1 mM EDTA and low-speed centrifugation. Cell pellets were then lysed at 4 °C for 30 min in a buffer containing 20 mM Tris–HCl, pH 7.4, 150 mM NaCl, 5 mM EDTA, 5% (v/v) glycerol, 10 mM NP-40 and protease inhibitor tablets (Roche)^61^. The supernatant fraction was obtained by centrifugation at 15,000 × g for 10 min at 4 °C and then stored at −80 °C. Protein concentration was determined by the Bio-Rad protein assay (Bio-Rad, Milan, Italy).

### Western blot analysis

For Western blot analysis, samples containing 30–50 μg of proteins were loaded on 10% SDS–PAGE and transferred to nitrocellulose membranes^62^,^63^. After blocking at room temperature in milk buffer [1 × PBS, 5–10% (w/v) non-fat dry milk, 0.2% (v/v) Tween-20], the membranes were incubated at 4 °C overnight with: 1:500 rabbit polyclonal antibody to human caspase-8 (Santa Cruz Biotechnology, Santa Cruz, CA); 1:500 mouse monoclonal antibody to human caspase-9 (Santa Cruz Biotechnology); 1:500 rabbit polyclonal antibody to human caspase-3 (Santa Cruz Biotechnology); 1:500 rabbit polyclonal antibody to human Bcl-2 (26 kDa) (Santa Cruz Biotechnology); 1:250 rabbit polyclonal antibody to human Bax (Santa Cruz Biotechnology, Santa Cruz). Subsequently, the membranes were incubated with peroxidase-conjugated goat anti-rabbit IgG, or with peroxidase-conjugated goat anti-mouse IgG + IgM (all the secondary antibodies were purchased from Jackson ImmunoResearch Laboratories). The resulting immunocomplexes were visualized by the ECL chemoluminescence method (ECL, Amersham Biosciences Little Chalfont, Buckinghamshire, UK) and analyzed by an imaging system (ImageQuantTM400, GE Healthcare Life Sciences)^64^. Densitometric analysis was conducted using the GS-800 imaging densitometer (Bio-Rad)^65^. Normalization of results was ensured by incubating the nitrocellulose membranes in parallel with the tubulin antibody (Sigma-Aldrich).

### Statistical Analysis

All data were presented as mean values ± SEM. The statistical analysis was performed using Graph-Pad Prism (Graph-Pad software Inc., San Diego, CA) and ANOVA test for multiple comparisons was performed followed by Bonferroni’s test.

## Additional Information

**How to cite this article**: Irace, C. *et al*. Antiproliferative effects of ruthenium-based nucleolipidic nanoaggregates in human models of breast cancer *in vitro*: insights into their mode of action. *Sci. Rep.* **7**, 45236; doi: 10.1038/srep45236 (2017).

**Publisher's note:** Springer Nature remains neutral with regard to jurisdictional claims in published maps and institutional affiliations.

## Figures and Tables

**Table 1:** IC_50_ values (μM) relative to the specified POPC and DOTAP ruthenium-containing liposomes, and to cisplatin (*c*DDP), used as cytotoxic reference drug, and to the naked AziRu complex in the listed breast cancer cell lines following 48 h of incubation.

Cell lines	IC_50_ (μM ± SEM)									
	ToThyRu/POPC		DoHuRu/POPC		ToThyRu/DOTAP		DoHuRu/DOTAP		AziRu	*c*DDP
	Total liposome	Ru	Total liposome	Ru	Total liposome	Ru	Total liposome	Ru		
MCF-7	185 ± 0.1	27.8 ± 0.1	126 ± 0.1	18.9 ± 0.1	74.6 ± 0.1	10.1 ± 0.1	67.3 ± 0.2	10.3 ± 0.2	>250	17 ± 1
MDA-MB-231	239 ± 3	35.8 ± 3	98 ± 1	14.7 ± 1	79.5 ± 0.2	10.8 ± 0.2	81 ± 0.3	12.1 ± 0.3	>250	19 ± 1.5
MDA-MB-436	>250	>37.5	245 ± 1	36.7 ± 1	110 ± 0.2	15.0 ± 0.2	130.9 ± 0.2	20.0 ± 0.2	>250	NA
MDA-MB-468	111.1 ± 0.1	15.7 ± 0.1	136 ± 0.8	20.4 ± 0.8	107.8 ± 0.1	14.7 ± 0.1	95 ± 0.1	14.2 ± 0.1	>250	24 ± 1
CG-5	138 ± 0.2	19.4 ± 0.2	204 ± 0.8	30.6 ± 0.8	30.1 ± 0.2	4.1 ± 0.2	21.5 ± 0.2	3.3 ± 0.2	>250	NA

The ruthenium IC_50_ values corresponding to the effective metal concentration (15% mol/mol) carried by each nanoaggregate are reported as mean values ± SEM (*n* = 30). (NA = not assessed).
